# Structural, Vibrational, and Magnetic Characterization of Orthoferrite LaFeO_3_ Ceramic Prepared by Reaction Flash Sintering

**DOI:** 10.3390/ma16031019

**Published:** 2023-01-22

**Authors:** Alejandro F. Manchón-Gordón, Pedro E. Sánchez-Jiménez, Javier S. Blázquez, Antonio Perejón, Luis A. Pérez-Maqueda

**Affiliations:** 1 Instituto de Ciencia de Materiales de Sevilla, CSIC-Universidad de Sevilla, C. Américo Vespucio 49, 41092 Sevilla, Spain; 2 Departamento de Química Inorgánica, Facultad de Química, Universidad de Sevilla, 41012 Sevilla, Spain; 3 Dpto. Física de la Materia Condensada, ICMSE-CSIC, Universidad de Sevilla, P.O. Box 1065, 41080 Sevilla, Spain

**Keywords:** reactive flash sintering, lanthanum ferrite, perovskite structure, Mössbauer spectroscopy, magnetic properties

## Abstract

LaFeO_3_ perovskite ceramics have been prepared via reaction flash technique using Fe_2_O_3_ and La_2_O_3_ as precursors. The obtained pellets have been investigated using several techniques. The formation of LaFeO_3_ has been clearly confirmed by X-ray diffraction. The scanning electron microscopy micrographs have shown the microporous character of the obtained pellets due to the low temperature and dwell time used in the synthesis process (10 min at 1173 K). The orthorhombic-rhombohedral phase transition has been observed at approximately 1273 K in differential thermal analysis measurements, which also allows us to determine the Néel temperature at 742 K. The fitted Mössbauer spectra exposed the presence of a single sextet ascribed to the Fe^+3^ ions in the tetrahedral site. Finally, magnetic measurements at room temperature indicate the antiferromagnetic character of the sample.

## 1. Introduction

Oxides with the AFeO_3_ (A = rare-earth) perovskite structure (the so-called orthoferrites) have been widely studied for their interesting properties and potential applications, such as catalysts [1] and gas sensors [2]. Among them, lanthanum orthoferrite (LaFeO_3_) has been extensively analyzed because it exhibits ferroelectric and ferromagnetic properties [3], similar to those found in BiFeO_3_-based compounds [4]. In particular, LaFeO_3_ and LaFeO_3_-based compounds are p-type semiconductors with outstanding physical and chemical properties [5], which have been employed as sensors [6], electrode materials in solid fuel cells [7] and photocatalysts [8].

LaFeO_3_ crystallizes in an orthorhombically distorted perovskite structure with the *Pnma* space group and is a canted antiferromagnetic material, because of superexchange interaction between neighboring Fe^3+^ ions. This compound exhibits a Néel temperature at $T_{N}\sim$740 K [9], which is the highest in the orthoferrite family. The interesting magnetic properties of this family of compounds have led them to be proposed, very recently, as promising candidates in the field of magnetic refrigeration [10] and as potential fillers for polymer nanocomposite magnets [11].

The performance of LaFeO_3_ commonly is subordinated to its structure, particle shape, and size. Consequently, the synthesis and processing methods employed in its preparation have a great influence on the resulting properties [12,13,14]. In this sense, diverse methods for the preparation of perovskite-type LaFeO_3_ have been described, based on the targeted applications [15]. This material is generally prepared by solid-state reaction technique [16], which commonly leads to the presence of secondary phases in ferrites [17]. Moreover, it often requires various heating and grinding steps to guarantee the homogeneous mixing of the oxide precursors employed [18,19,20], with posterior calcination at temperatures higher than 1773 K for 6 h [3]. Alternative synthesis techniques, such as combustion [21], mechanochemistry [22] and soft-chemistry [23] have been proposed to circumvent those limitations and facilitate the preparation of lanthanum ferrites free of secondary phases and nanostructured. Yet, high sintering temperatures are still required to prepare fully dense LaFeO_3_ specimens due to poor sinterability [13]. Spark Plasma Sintering (SPS) can be used to reduce sintering temperature down to 923 K but at the expense of modification in the electrical properties due to the highly reducing environment in the SPS chamber [24]. A recently proposed current-assisted sintering technique, Flash Sintering (FS), has demonstrated the capability to significantly reduce the sintering temperature of ceramic materials [25]. In this technique, first proposed in 2010, heat and a modest electric field are applied simultaneously to the sample, through which a certain current density flow. At a certain temperature, which depends on several factors such as the applied electric field and the intrinsic conductivity of the material, a dramatic decrease in the electrical resistance of the specimens occurs, generally accompanied by rapid densification [26]. Since its proposal, FS has grabbed the interest of the scientific community, which has devoted an extraordinary effort to the development of the technique. For example, this technique has been extended to 3D complex-shaped specimens that go beyond the typical dog bones or disks, using a multiphase power supply and multiple electrodes [27]. Furthermore, Gil-González et al. demonstrated the feasibility of employing FS to produce dense and single-phase complex oxides in a single step from their precursors [28]. This new methodology, which has been denominated Reactive Flash Sintering (RFS), has been recently employed in the preparation of an extensive range of materials, such as ferrites [29,30], ferroelectric materials [31,32], ceramic composites [33,34], high-entropy oxides [35,36,37] and solid electrolytes [38,39]. FS and RFS techniques have been also employed for the preparation of LaFeO_3_-based compounds. For example, it has been achieved the sintering of La_0.6_Sr_0.4_Co_0.2_Fe_0.8_O_3_ at temperatures lower than 373 K [40] and the co-sintering of a lanthanum strontium cobalt ferrite/Gd-doped ceria bilayer structure by FS [41]. It has been also reported the preparation of lanthanum strontium cobalt ferrite via reaction flash light sintering, in which the material is exposed to a xenon arc lamp [42]. However, to the best of our knowledge, the preparation of phase-pure LaFeO_3_ perovskite by RFS has not been reported.

The purpose of this work is to further demonstrate the capability of RFS for the preparation of multiferroic and magnetic materials which are hard to prepare by conventional techniques. Thus, we show that phase-pure LaFeO_3_ pellets can be indeed prepared by this technique. The crystal structure, microstructure, thermal, vibrational, and magnetic properties of the obtained pellets were characterized. Mössbauer spectroscopy has been used to obtain selective information about the iron ions, such as the oxidation state, coordination, and the existence of impurity phases in the prepared LaFeO_3_ pellets.

## 2. Materials and Methods

LaFeO_3_ was synthesized from commercially available α-Fe_2_O_3_ (Sigma Aldrich, Darmstadt, Germany; <5 μm, ≥99% purity) and La_2_O_3_ (Sigma Aldrich, Darmstadt, Germany; ≥99.9% purity) powders. The oxides were dried in a warming oven for 0.5 h to remove any moisture and mixed by ball milling in stoichiometric proportions. To prepare the pellets, approximately 0.8 g of the precursor powder was mixed with a polyvinyl alcohol binder solution and uniaxially pressed into dog-bone shapes under a pressure of 500 MPa for 3 min. Subsequently, the obtained dog bones were pre-heated at 723 K for 0.5 h in the air to remove the binder prior to the RFS experiment. Electrodes consisted of two platinum wires attached to the handles of the dog bone and connected to a 1500 W DC power supply (EA-PSI 9760-06 DT). The RFS experiment was carried out in a tubular furnace keeping constant the temperature while the intensity current limit was progressively increased at 10 mA s^−1^. Once the selected intensity current limit was reached, the flash state was maintained for 10 min. After this time, the furnace and power supply were shut down.

The preparation of LaFeO_3_ was followed, at room temperature, by X-ray diffraction technique using a Rigaku MiniFlex diffractometer with Cu-Kα radiation, λ = 1.5406 Å. Phase transition temperatures were determined from differential scanning calorimetry (DSC) measurements, using a simultaneous TG/DSC (Q650 SDT, TA Instruments) under a nitrogen flow and a heating rate of 10 K/min. Scanning electron microscopy (SEM) was employed to study the microstructure of the obtained pellets using an FEI Teneo microscope. To do that, the surface morphology of the fracture of the samples was directly observed after a block was cut from the original pellet.

^57^Fe Mössbauer spectroscopy was carried out at room temperature using a ^57^Co(Rh) source in transmission geometry. Samples were measured as powders by mechanically grinding the pellets. NORMOS program [43] has been employed to determine hyperfine parameters. As it is conventionally done, isomer shift is given relative to an α-Fe foil. Raman spectroscopy was measured on the fracture surfaces of the samples using a dispersive microscope Raman Horiva Jobin Yvon LabRam HR800 operating at 532.1 nm. At least ten spectra were acquired from each sample.

Magnetic properties were characterized in a Quantum Design Physical Properties Measurement System, PPMS, using the vibrating sample magnetometer standard option. Hysteresis loops at room temperature were carried out using a maximum applied magnetic field of 5 T.

## 3. Results and Discussion

**Figure 1:**
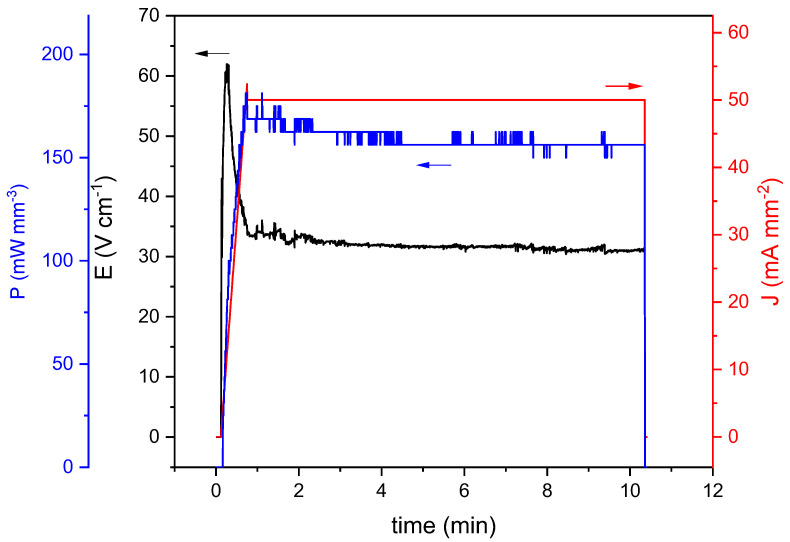
Evolution of reaction flash parameters, electric field, power density dissipation, and current density limit, as a function of time during the reactive flash process.

The preparation of the desired product by reactive flash sintering requires meticulous control of the experimental conditions [29] due to the thermal management during the flash event, which results in sample heterogeneities such as abnormal grain growth, preferential current paths, and local melting [44]. To avoid these problems, typical of conventional flash experiments in which the electric field is kept constant while the temperature is linearly increased, the use of current density ramps has been proposed [45]. This variation of the technique allows increasing the current density circulating through the material linearly with time while the applied voltage is automatically adjusted. Thus, it enables to pre-define the onset temperature, i.e., the temperature at which the flash event takes place. In this work, we have employed current density ramps to explore if it is viable to prepare single-phase LaFeO_3_ orthorferrite via reaction flash sintering. Figure 1 depicts the variation in typical electrical parameters involved in a flash experiment with time during the RFS process when a current density ramp is employed. A sharp increase in the electric field at the beginning of the process is clearly observed, which is necessary to follow the imposed current density ramp. Then, the electric field decreases to a stable value when the preset current density value is reached. Similar behavior can be observed in the case of the power density dissipated, P=JE, where J is the current density and E the electric field.

**Figure 2:**
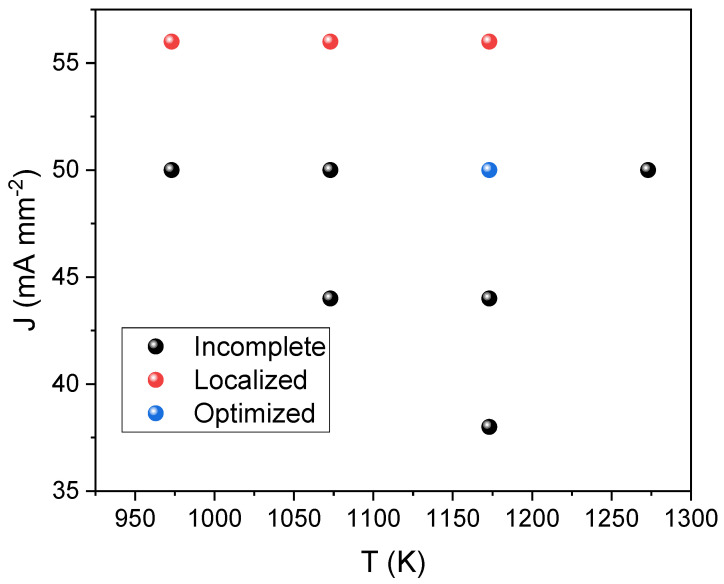
Experimental conditions tested in the reactive flash process of La_2_O_3_ and Fe_2_O_3_, in terms of onset temperature and current density limits (flash event holding 10 min).

Several experiments have been performed to determine the optimum flash conditions, using different combinations of onset temperature and current density limits. Obtained results have been depicted in Figure 2. As can be observed, only under very specific conditions, 1173 K and 50 mA mm^−2^, phase-pure samples can be prepared. In any other case, the explored experimental conditions result in incomplete reactions, as will be discussed below, or in samples exhibiting current localization or hot spots. The latter involves the formation of preferential paths for the current flow that results in highly heterogeneous specimens [29]. 

**Figure 3:**
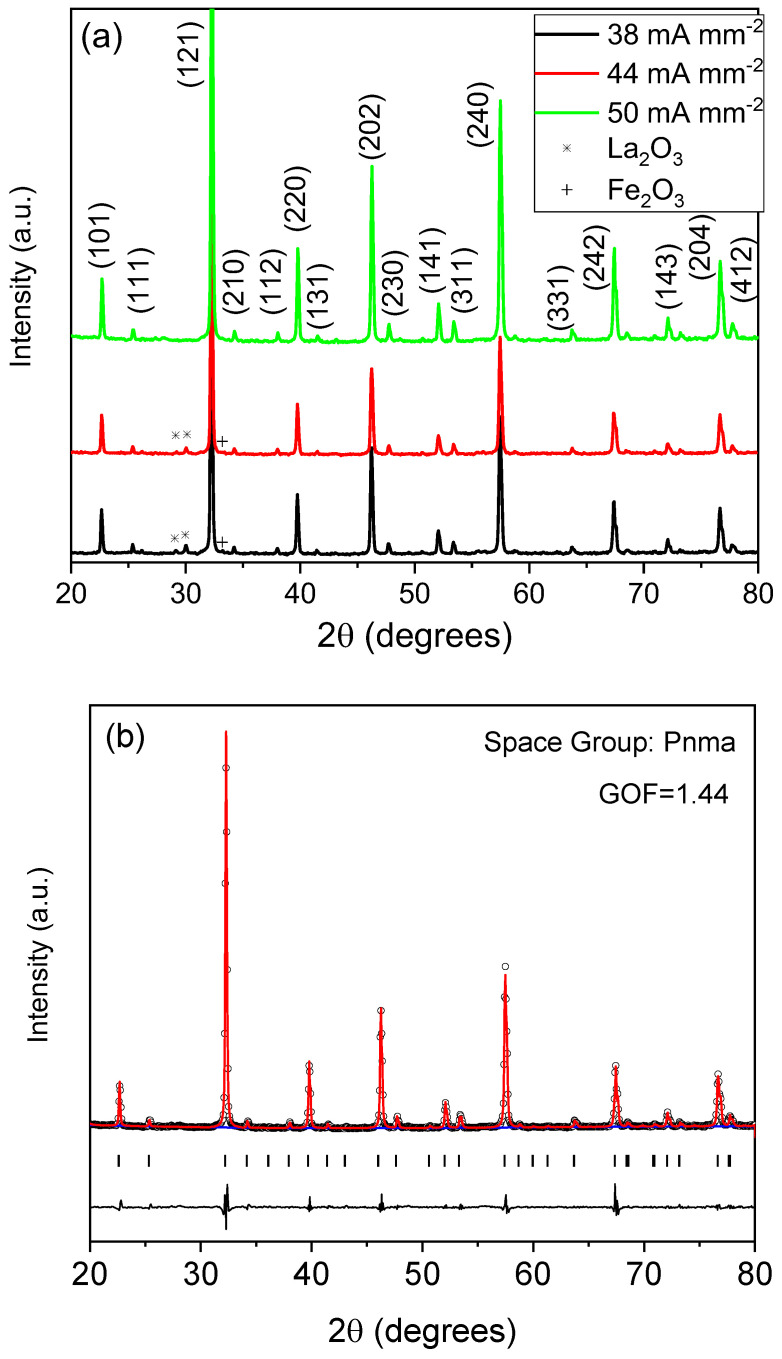
(**a**) XRD patterns of the RFS-ed samples under different current density limits at 1173 K. The indexed peaks correspond to the main diffraction peaks of the LaFeO_3_ compound (space group Pnma). The traces of the precursors have been also indicated. (**b**) Le Bail refinement of the XRD pattern corresponding to the phase-pure specimen.

The crystal structure and composition of the prepared LaFeO_3_ pellets were studied by X-ray diffraction at room temperature. The XRD profiles of specimens flashed at 1173 K with different current density limits are presented in Figure 3. As can be observed, if the maximum current density is set to 38 mA mm^−2^, traces of the precursors (Fe_2_O_3_ and La_2_O_3_) are detected alongside the perovskite structure. This fact indicates that insufficient energy has been supplied to produce the desired LaFeO_3_ phase. By increasing the current density, the amount of precursors after the RFS process decreases, and by further increasing the maximum current density to 50 mA mm^−2^, no traces of the precursors were detected, and phase-pure LaFeO_3_ were obtained.

Le Bail refinement of the XRD pattern corresponding to the phase-pure specimen has been depicted in Figure 3b. All peaks could only be indexed by the Pnma space group. The obtained lattice parameters were *a* = 5.5603(2) Å, *b* = 7.8447(8) Å, *c* = 5.5438(4) Å and the crystal size was 119(2) nm. The obtained values of the crystallographic parameters are similar to those obtained for high-quality LaFeO_3_ samples [46].

**Figure 4:**
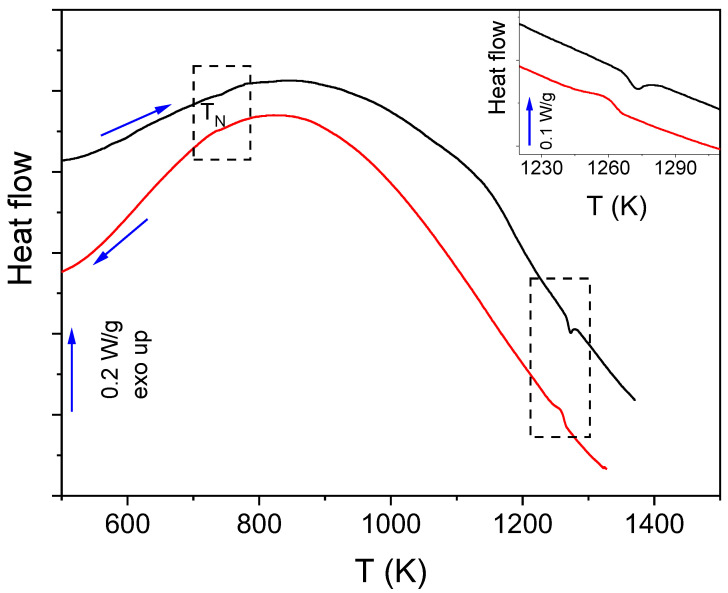
DTA scans at 10 K min^−1^ for phase-pure LaFeO_3_ obtained by RFS. Inset shows a horizontally zoomed plot in a limited range of temperatures (black and red lines correspond to heating and cooling, respectively).

The phase transitions of the prepared samples were studied by differential scanning calorimetry. Figure 4 shows the DTA scans at 10 K min^−1^ of the phase-pure sample both on heating and cooling. The weak endothermic peak around 742 K corresponds to the Néel temperature, $T_{N}$, which indicates the antiferromagnetic-paramagnetic transition. The second endothermic peak, much more intense, that appears around 1262 K is associated with the orthorhombic-rhombohedral phase transition. No more peaks, which could have revealed the existence of impurities, have been detected. In fact, both $T_{N}$ and the temperature of the phase transition is in agreement with those reported in the literature for high-quality LaFeO_3_ samples [13,47].

**Figure 5:**
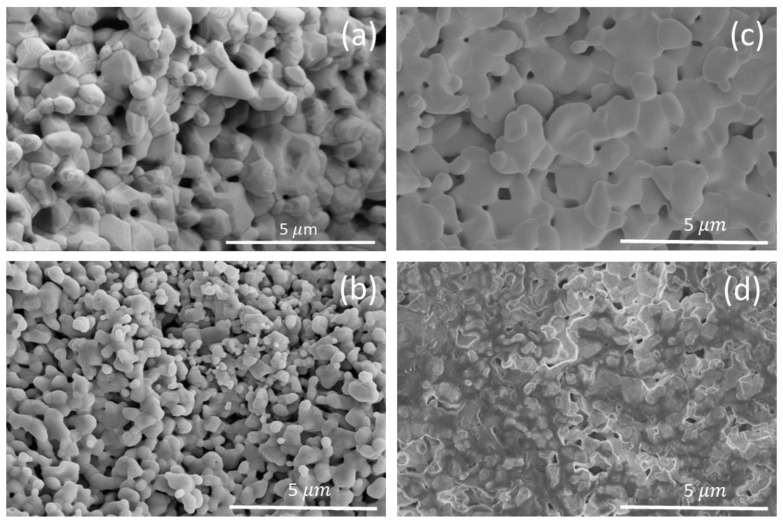
SEM micrographs of cross-section fractures of the specimens obtained using a maximum current density of 50 mA mm^−2^ at (**a**) 1173 K (optimized conditions), (**b**) 1073 K, (**c**) 1273 K. (**d**) Micrograph that shows the effect of current localization (56 mA mm^−2^ at 1173 K).

Figure 5a depicts representative SEM images of the cross-section fracture of the phase-pure LaFeO_3_ specimen. The micrograph exhibits an inhomogeneous distribution of grains. Thus, the grains are of irregular shape, and the sizes are lower than 1 μm. In fact, the small grain size in RFS-ed samples is typical of ferrites produced by this technique due to the low sintering temperature and time needed, which limits grain growth [30,48,49]. The observed microstructure is consistent with a not totally sintered material since some micropores have been clearly detected. However, it is significant the relative density is around 80% (estimated from Archimedes method) considering the time and temperature employed in the experiments carried out in this work. In fact, it has been reported that obtaining relative densities around 90% in LaFeO_3_ requires long dwell times and high temperatures (1673 K and 10 h) [13], much higher than the furnace temperature of 1173 K required by RFS. This entails substantial energy saving [49]. In this sense, although the reaction of the specimen prepared at a furnace temperature of 1273 K was incomplete, the sample exhibits higher grain size and density (Figure 5c). On the contrary, lower temperatures induce lower grain size and density (Figure 5b). In Figure 5d, the effect of the current localization on the morphology of the specimen can be observed.

The characteristic crystal structure of the orthoferrite compounds makes Raman spectroscopy an extremely useful technique to obtain relevant information on various dynamical processes involving phonons, charge carriers, and spins [50,51,52]. On the other hand, Raman spectroscopy is known to be a powerful technique to determine the structure distortion and oxygen motion of perovskite-type materials [53]. Although no Raman modes would be expected in the case of an ideal cubic ABO_3_ perovskite structure, the characteristic distortion of the orthorhombic structure induces the activation of Raman vibration modes, in which atoms occupy four non-equivalent positions and only B atoms are situated at the center of symmetry in the corner-sharing BO_6_ octahedral network [54,55].

**Figure 6:**
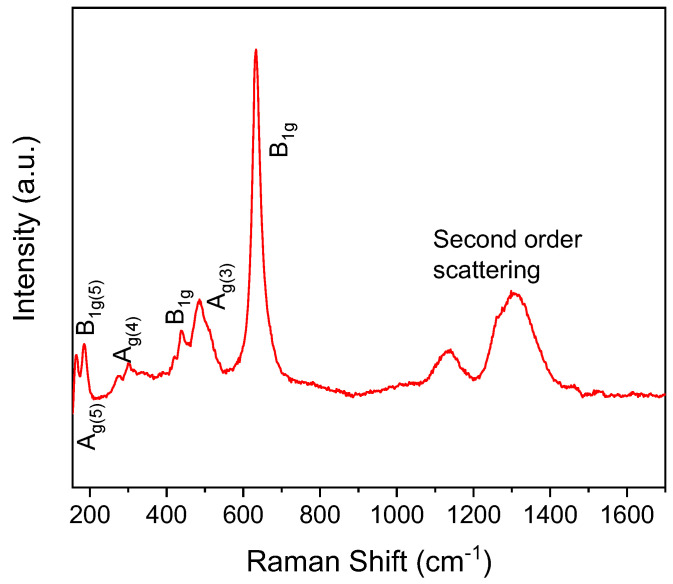
Raman spectrum of phase-pure LaFeO_3_ at room temperature.

Figure 6 shows the Raman spectrum of the phase-pure LaFeO_3_ sample taken at room temperature. The position of the observed modes has been collected in Table 1, which is in good agreement with those reported in the literature [53,56,57]. In LaFeO_3_ ceramics, Raman modes lower than 200 cm^−1^ are ascribed to the deformation motivated by displacement of La cations. The vibrational modes close to 300 cm^−1^ include several components and it is ascribed to the FeO_6_ octahedral tilt modes, while the mode around 450 cm^−1^ is associated with the oxygen octahedral bending vibrations. Raman mode centered around 520 cm^−1^ belongs to A_g_ symmetry and the mode observed at 630 cm^−1^ reflects oxygen stretching vibrations. Finally, the broad features found above 1000 cm^−1^ are attributed to second-order scattering [21,57].

**Figure 7:**
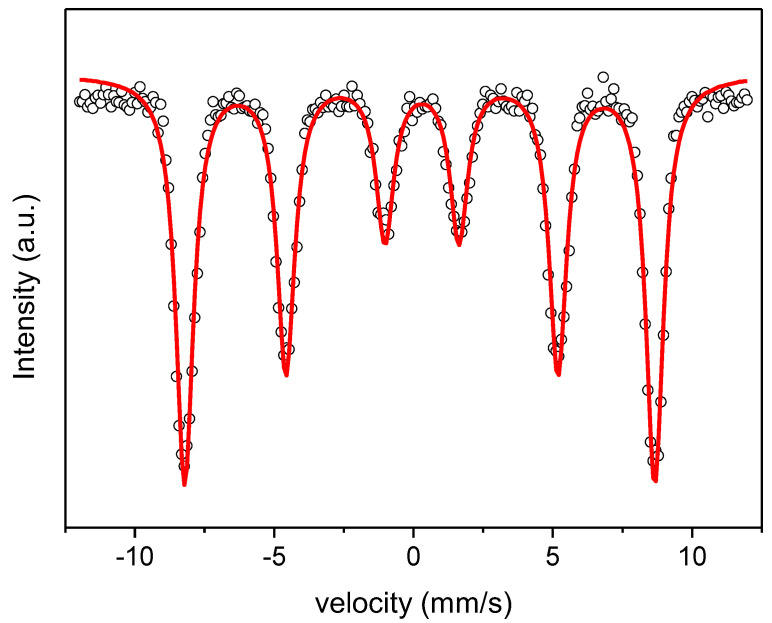
Experimental room temperature Mössbauer spectrum (symbols) and model fitting (lines) of the resulting LaFeO_3_ pellet obtained after the reaction flash sintering process.

Figure 7 depicts the Mössbauer spectrum of the phase-pure LaFeO_3_ specimen taken at room temperature. It can be observed that the spectrum exhibits a single sharp sextet, which has been fitted with a single sextet. The hyperfine parameters denote that iron in the LaFeO_3_ compound presents a valence state 3+, indicated by the obtained values of isomer shift, δ = 0.25(2) mm s^−1^. In fact, a valence state 2+ is indicated for values of δ > 0.9 mm s^−1^ [58]. On the other hand, the high magnetic hyperfine field obtained, $B_{hf}$ = 52.2(3) T, indicates a strong Fe-Fe coupling, suggesting an antiferromagnetic behavior of the studied compound, as will be discussed below. Additionally, the small value of the quadrupole splitting, QS = −0.08(2) mm s^−1^, suggests the distortion of the octahedral arrangement of oxygens [56], in agreement with the Raman results. No other contributions have been detected by Mössbauer spectroscopy, indicating that the sample is phase-pure, in agreement with the XRD and DSC results presented above. The obtained values of hyperfine parameters are in agreement with those described in the literature [56,59].

**Figure 8:**
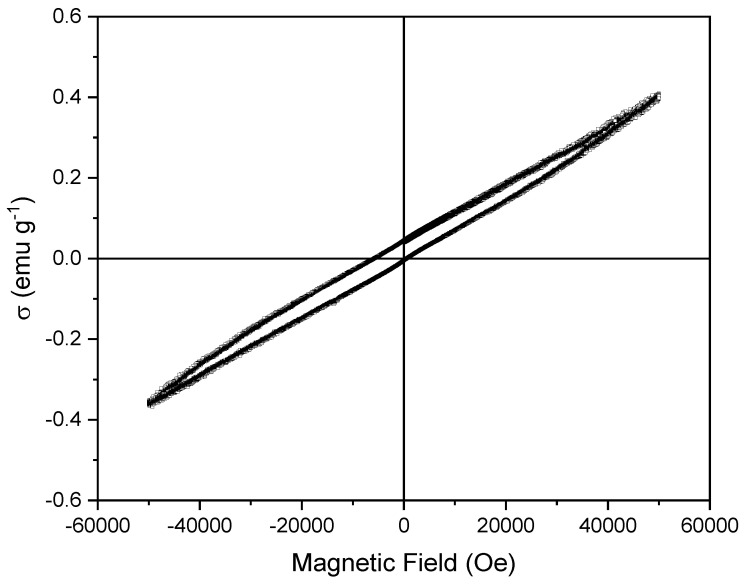
Magnetic hysteresis loop of the resulting LaFeO_3_ pellet obtained after the reaction flash sintering process.

Figure 8 depicts the hysteresis loop of the LaFeO_3_ phase-pure sample taken at room temperature under an applied magnetic field ranging between −5 and 5 T. As it was expected, the studied specimen exhibits an almost linear field dependence of magnetization, typical of antiferromagnetic materials, which indicates that the magnetization (or remnant magnetization) is almost zero, confirming the antiferromagnetic character of this sample caused by strong Fe-Fe couplings [60]. However, some hysteresis can be observed, which could be related to the presence of vacancies, typical of samples prepared by flash sintering [61,62], which affect the Fe-Fe couplings. In fact, the absolute magnetization of the sample is less than 0.5 emu g^−1^ even at high applied fields. The observed unsaturation of the sample at the maximum applied field is due to the inconsistent direction of the moments, which are also signals of antiferromagnetism.

## 4. Conclusions

Reaction flash sintering has been successfully employed to produce phase-pure orthorhombic LaFeO_3_ compound in a single step in just 10 min holding time. The X-ray diffraction patterns showed that the obtained sample was a single phase with an orthorhombic structure, confirmed by Mössbauer and Raman spectroscopies. Moreover, Mössbauer fittings reveal a strong Fe-Fe coupling characterized by a high value of the hyperfine field. These results suggest an antiferromagnetic behavior of the studied compound, as it has been checked by magnetic measurements at room temperature. The studied compound exhibits two phase transitions in the temperature range analyzed, as it was determined by differential thermal analysis. These transitions correspond to Néel temperature and the orthorhombic-rhombohedral phase transition.

The obtained results are largely in line with previous results of high-quality LaFeO_3_ samples prepared by other techniques that require more time and energy_._ Moreover, reaction flash sintering is a time and energy-saving route as compared to conventional solid-state reaction. Thus, the results presented in this work expose the potential of reaction flash sintering for the synthesis and sintering of multiferroic materials in a greener way.

Further studies are welcomed to analyze the effect of time in which the flash event was maintained on the relative density achieved and the grain size. In this sense, the microporous character of the obtained samples makes them candidates for possible applications in photocatalysis. Moreover, the possibility of reducing the problem associated with the localization of the current by AC measurements should be also explored in future studies.

## Figures and Tables

**Table 1:** Position of Raman modes and their assignments for the phase-pure LaFeO_3_ compound prepared by RFS.

Raman Modes	Raman Shift (cm^−1^)
B_1g(5)_	166
A_g(5)_	188
A_g(4)_	274
	301
	334
B_1g(3)_	438
B_3g(3)_	486
A_g(3)_	515
B_1g_	631
Second order scattering	1133
	1305

## Data Availability

Data will be available on request.
